# Combining the unequal variance signal detection model with the health belief model to optimize shared decision making in tinnitus patients: part 2—patient profiling

**DOI:** 10.3389/fnins.2025.1466354

**Published:** 2025-05-19

**Authors:** Zsófia Zs Lehóczky, Adriana L. Smit, Sarah Kaldenbach, Arnold Lieftink, Huib Versnel, Robert J. Stokroos, Alexander E. Hoetink

**Affiliations:** ^1^Department of Otorhinolaryngology - Head and Neck Surgery, University Medical Center Utrecht, Utrecht, Netherlands; ^2^UMC Utrecht Brain Center, Utrecht, Netherlands; ^3^Dutch Foundation for the Deaf and Hard of Hearing Child (NSDSK), Amsterdam, Netherlands

**Keywords:** tinnitus, psychophysics, shared decision making, sound therapy, cognitive behavioral therapy, hearing loss, signal detection theory, health belief model

## Abstract

**Introduction:**

Tinnitus affects approximately 14% of the population. Its symptomatology is versatile, ranging from mild annoyance to anxiety and depression. Current multidisciplinary treatments (psychological, audiological, and combinations) focus on impact reduction and acceptance. Shared decision making (SDM) promotes patients and health care professionals making treatment choices together based on the best available evidence. In the case of professional equipoise (no clear clinical evidence for superiority of a treatment), knowledge about individual factors influencing the outcome of patient decisions can be of utmost importance in informing the SDM process.

**Methods:**

A statistical model that was developed in previous work to analyze tinnitus patient decisions, was extended to analyze how patient characteristics on sex, age, and laterality of tinnitus affect the accuracy and utility of decisions concerning audiological care and cognitive behavioral therapy (CBT) based psychosocial counseling. For each group, we calculated Receiver-Operator-Characteristic curves and likelihood ratio curves as function of hearing loss and pre-treatment tinnitus impact to assess accuracy and utility of decisions for audiological care and CBT-based counseling, respectively.

**Results:**

The largest effect was found for sex differences. The results indicated that males used a strict decision criterion when deciding about psychosocial counseling, while females used a strict decision criterion for decisions about audiological care. The likelihood ratios of a successful treatment versus unsuccessful treatment are smaller than 1 for psychosocial counseling for females and for audiological care for males. The likelihood ratios of success are approximately 2 and almost 7 for audiological care for females and psychosocial counseling for males, respectively. For age differences, older participants adopted a more lenient decision criterion for audiological care across most of the hearing loss range, while younger participants adopt a stricter decision criterion up to hearing losses of approximately 75 dB(HL). For psychosocial counseling, older participants adopted an unbiased criterion and younger participants a strict decision criterion. For the younger group, psychological counseling seems more likely to be successful compared to the older group. When considering laterality, for audiological care the group with unilateral tinnitus adopted a strict decision criterion for the whole range of hearing loss, while the group with bilateral tinnitus adopted a strict decision criterion for hearing losses above approximately 70 dB(HL). For decisions about psychosocial counseling, the unilateral tinnitus group adopt a strict decision criterion for baseline THI-scores between approximately 25 and 90 points. The bilateral tinnitus group adopted an unbiased to strict decision criterion for psychosocial counseling for the entire baseline THI-score range.

**Discussion:**

These findings underscore the importance of personalized treatment approaches based on specific patient characteristics and the need for further research to test and improve these findings. Especially males may be more strongly advised to take up psychosocial counseling and females may be more strongly advised to take up audiological care. For age and laterality, the results are more diffuse.

## 1 Introduction

The prevalence of chronic tinnitus ranges from 4.7% to 19.3%, depending on demographic and geographic factors (Jarach et al., 2022; Knipper et al., 2020). Based on previous research it is estimated that in 40% of the cases the cause leading to the onset of chronic subjective tinnitus is unclear to the patient (Henry et al., 2005). There is a range of known factors that can lead to the development of the condition, e.g., hearing loss, usage of medication, head or neck injuries, or noise exposure. In clinical practice, chronic subjective tinnitus is usually attributed to more than one cause (Knipper et al., 2020). Its versatile symptomatology consists of and causes psychological distress—in several cases, disorders—ranging from mild annoyance to anxiety and depression (Baguley et al., 2013; Sedley et al., 2016). In general, the condition has fundamental negative impacts on patients’ quality of life that are difficult to objectively assess due to different individual variations in development and experience (De Ridder et al., 2014; Henry et al., 2005). However, there are sensitive tools available to quantify the impact of tinnitus on quality of life, such as the widely used tinnitus handicap inventory (THI), which is a 25-item questionnaire assessing the impact of tinnitus on functional, emotional and physical aspects.

Since chronic subjective tinnitus constitutes physiological and psychological factors, professionals in the field support multidisciplinary approaches to address and reduce the symptoms (Baguley et al., 2013). There is a lack of reliable and consistent evidence on the efficacy of the individual and combination treatments, such as audiological care and psychological treatment. This is due to different study designs and lack of reproducible results (Fuller et al., 2020; Hoare et al., 2014; Sereda et al., 2018). This lack of evidence concerning tinnitus treatment options gives rise to a general professional equipoise, i.e., uncertainty about the comparative power of available treatments due to heterogeneous and inconclusive clinical results on their benefits (Elwyn et al., 2000). Currently, standard tinnitus management comprises psychoeducation, audiological treatments, and psychological therapies (Baguley et al., 2013; Makar et al., 2017). Widely used audiological treatments are different sound therapy approaches, such as sound enrichment and hearing aids. These, respectively, aim to replace the disturbing perception of tinnitus with another more pleasant sound or to reduce the sensitivity of the auditory system by restoring afferent input (Searchfield, 2021). The most prominent and common psychological treatments are cognitive behavioral therapy (CBT) based methods since they show higher effectiveness in decreasing distress and mental disturbance associated with tinnitus in comparison to other methods (Makar et al., 2017; Marks et al., 2019). These treatments help the individual understand and accept the condition, improving coping through acceptance (Makar et al., 2017). Marks et al. (2019) claim that a biopsychological approach offers more comprehensive care and reduces the burden of tinnitus more effectively. Despite the extensive literature, there is little consensus on treatment effectiveness for chronic tinnitus (Fuller et al., 2020; Marks et al., 2019). This emphasizes that incorporating individual factors in treatment planning is essential in improving outcomes.

Most research highlights genetic, cognitive, and behavioral variations in tinnitus (Mazurek et al., 2019). Many studies have focused on biological sex, age and the symptomatology of tinnitus as individual factors (Henry et al., 2005; Sedley et al., 2016; Krauss et al., 2016; Oosterloo et al., 2020; Schilling et al., 2023). Understanding patient behavior is of utmost importance in successfully aligning tinnitus treatment to the patient needs and to establish a sensitive guidance in the decision-making process. Several studies analyzed help-seeking behavior, providing insights into factors influencing patients’ attitude toward professional help and their treatment choices (Cooper et al., 2019; Güney et al., 2024; Lim et al., 2019; Nagai et al., 2023; Rademaker et al., 2021; Teo et al., 2022; Vestergaard Knudsen et al., 2010). Help-seeking is found to be most positively correlated with self-perceived hearing limitation (Rademaker et al., 2021; Vestergaard Knudsen et al., 2010) and tinnitus impact on quality of life (Rademaker et al., 2021). Most tinnitus patients also experience hearing loss (Krauss et al., 2016; Schilling et al., 2023; Sedley et al., 2016) and consequent life quality deterioration (Rademaker et al., 2021). Hence hearing loss and baseline THI-score are key drivers in decisions about audiological and psychological help (Hoetink et al., 2024). In the following we will summarize the literature on how the factors sex, age and tinnitus laterality may influence the experience of tinnitus. Also, the literature on the effect of these factors on treatment preferences will be narratively reviewed.

Sex differences in the experience of tinnitus and help-seeking behavior are complex and influenced by various psychological, social, and cultural factors. For instance, women are more likely to exhibit emotional reactions, such as depressive states, to the diagnosis compared to men, suggesting a greater impact on their quality of life (Vanneste et al., 2012). A summary of the literature on help-seeking behavior by Güney et al. (2024) suggests that no consensus exists about the role of sex in help-seeking behavior, however, there are substantiated tendencies. For instance, there are several studies suggesting that females are generally more likely to seek help for their general and psychological issues than men (Güney et al., 2024; Lim et al., 2019). They are less likely, however, to seek professional assistance compared to men (Güney et al., 2024; Nagai et al., 2023). Instead, they turn to friends and family for advice, seeing help-seeking as a way of strengthening close relationships (Nagai et al., 2023). When the severity of the problem (e.g., depression) increases, both men and women were found to be less likely to seek professional help (Nagai et al., 2023). Cultural and societal factors, such as stigma, masculine ideals and less gender-sensitive therapy options have been reported to contribute to men’s hesitation in seeking psychological help (Cooper et al., 2019; Güney et al., 2024) despite experiencing higher rates of psychological struggles (Cooper et al., 2019; Liddon et al., 2018). For instance, female psychotherapists, if unaware of their preferences for treatment, can use methods that are incompatible with male patients’ needs, such as employing warm support instead of confrontation (Cooper et al., 2019). The inclination to avoid seeking help extends beyond psychological services to general health services as well (Güney et al., 2024). However, in a review of literature by Vestergaard Knudsen et al. (2010), sex is reported to be an irrelevant factor in audiological help seeking.

There are conflicting results in the literature concerning the role of age in experiencing tinnitus and seeking help for it. According to Oosterloo et al. (2020), aging is not associated with tinnitus. This suggests that, even though hearing impairment is a clear correlate of tinnitus (Krauss et al., 2016; Schilling et al., 2023; Sedley et al., 2016), age-related hearing loss is not. This may indicate that the brain can adapt to the gradual loss of hearing over time. This aligns with some findings in the literature. Vestergaard Knudsen et al. (2010) similarly found age to be an irrelevant factor in audiological help seeking. In case of psychological help-seeking Lim et al. (2019) found no difference in the healthcare-seeking rate between individuals aged below and above 50 years. On the other hand, Güney et al. (2024) found that age was a clear negative correlate, meaning that older people are less likely to ask for psychological help. Teo et al. (2022) also conclude that due to the complexity of societal barriers (e.g., ageism, financial troubles), older individuals may be less likely to seek professional help. This calls for their inclusion in decision-making to increase healthcare access.

Rademaker et al. (2021) found that help-seeking tinnitus patients exhibited higher tinnitus impact scores and experienced more severe and multifaceted tinnitus symptoms compared to non-help seekers. They found no differences between help-seekers and non-help seekers regarding side of tinnitus perception. This is contrary to earlier findings. Hallberg and Erlandsson (1993) looked at differences between help-seekers and non-help-seekers with tinnitus, finding differences in the laterality of the patient’s tinnitus, amongst others. Bilateral tinnitus was more prevalent among non-help-seekers, while unilateral tinnitus is more common in clinical populations. Furthermore, unilateral tinnitus seemed negatively associated with treatment outcomes (Hallberg and Erlandsson, 1993). We did not identify any studies specifically examining how the laterality of tinnitus influences the outcomes of psychological treatments.

Elwyn et al. (2010) and Elwyn et al. (2012) promote the importance that practices are adopted to the patient’s decision-making process and priorities. To assist a tinnitus patient effectively and sensitively in shared decision making (SDM), health care professionals have to understand the influence different personal factors may have on the individual’s preferences (Stiggelbout et al., 2012). In Part 1 of this study, we presented a model that combines principles from the health belief model (HBM) and signal detection theory (SDT) to map the drivers for tinnitus patient decisions for audiological care and CBT-based treatments. The findings reveal that hearing loss is the primary driver for choosing audiological care. The pre-treatment impact of tinnitus on quality of life, measured with the THI questionnaire, drives the preference for psychosocial counseling (Hoetink et al., 2024). We also identified differences in participant characteristic between the different treatment groups. In this part, we will further explore these dissimilarities. We will extend the model by stratifying according to sex, age, and tinnitus laterality, to study the accuracy and utility of SDM. In our exploratory study, the direction of the differences to be expected is unclear, in line with the heterogeneous results in the literature.

## 2 Materials and methods

### 2.1 Study design and setting

Data collection is described in detail in Part 1 (Hoetink et al., 2024). In summary, clinical data was collected by convenience sampling during a two-year period from an outpatient audiology clinic in Alkmaar, The Netherlands. Data collection included results of audiological assessments, demographic information and tinnitus characteristics.

### 2.2 Participants

**FIGURE 1:**
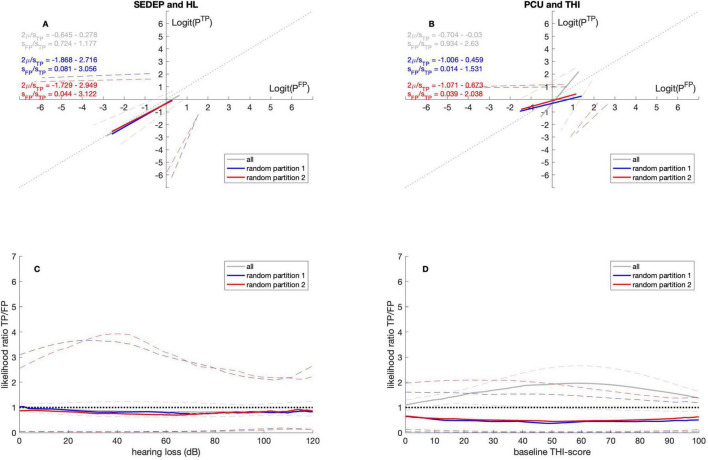
ROC curves **(A,B)** and likelihood ratio functions **(C,D)**. Gray lines for datasets with all participants, blue lines for datasets with random permutations of 72 out of 143 participants (random partition 1) and red lines for datasets with the remaining 71 participants (random partition 2). The solid lines denote the medians and the dashed lines the 2.5 percentiles and 97.5 percentiles, respectively. SEDEP, sound enrichment device evaluation period; HL, hearing loss; PCU, psychosocial counseling uptake; THI, tinnitus handicap inventory; TP, true positive; FP, false positive. See text for a discussion.

150 participants with subjective tinnitus were included based on the eligibility criteria for age (18 years and older), primary referral for tinnitus care, and no current use of a hearing aid, a sound generator, or a combination device. Five initially eligible participants were excluded due to either lack of informed consent (*n* = 4) or double entry (*n* = 1) during the study flow. The final number of participants was 145. For 2 participants information about their decision to take up psychosocial counseling was missing, leaving 143 participants for analysis. For more details, see the flow chart in Figure 1 of Part 1 (Hoetink et al., 2024). Ethical approval was given by the Medical Research Ethics Committee Noord-Holland (M010-34).

### 2.3 Treatment

Treatment options are extensively described in Part 1. Summarizing, each participant received psychoeducation as part of standard care. Psychoeducation provided information on the physiology of the ear, the process of hearing, and the different neurophysiologic and somatic causes of tinnitus. Also, the results of the audiological assessment were discussed. In addition, the effect of tinnitus on psychosocial functioning and physical wellbeing was discussed, along with the influence of emotions, stress, drugs and alcohol on the condition. Finally, the follow-up treatment options were detailed, which entailed audiological care (starting an evaluation period with hearing aids with an optional sound generator), taking up CBT-based psychosocial counseling, a combination of the two, or no follow-up treatment. All participants were offered the same follow-up treatment options.

### 2.4 Variables

The primary outcome measure was the change in tinnitus related quality of life measured by the THI questionnaire. Participants filled out the questionnaire after receiving informed consent but before the audiological assessment (baseline), and after finishing follow-up treatment. The response rate at baseline was 97%, and post-treatment 63%. Missing data was imputed based on observed values for a particular participant and the relations observed in the data for all other participants, see Part 1 for details. The clinically important change in THI-score was determined at 7 points, Zeman et al. (2011).

The variables sex (male/female), age (younger than 55 years/55 years and older), and laterality of tinnitus (unilateral/bilateral) were defined as grouping variables. These variables were included as they are the most discussed factors in individual variation of tinnitus in the literature. The following decision-driver and treatment combinations were assessed: hearing loss at 8 kHz at the participant’s worse-functioning ear and audiological care; baseline THI-score and psychological counseling. Continuous variables with a normal distribution are displayed as mean and standard deviation (SD), continuous non-normally distributed data are shown as median and interquartile range (IQR), and discrete variables are shown as number and percentage.

### 2.5 Data analysis

Data analysis was conducted using MATLAB^®^ software, R2024b (24.2.0.2712019). We used the statistical model based on signal detection theory that was constructed in Part 1 (Hoetink et al., 2024). For both combinations of driver and treatment (hearing loss and audiological care; baseline THI-score and psychosocial counseling) we estimated the parameters (μ,*s*) of the logistic distribution of the population of participants that experienced a change in THI-score of less than 7 points (which we will call a negative truth state, say T−) and the population of participants that experienced a change in THI-score of more than 7 points (which we will call a positive truth state, say *T*+). The model was extended by stratifying for the different grouping variables (sex, age and tinnitus laterality). Bayesian inference was used to estimate the parameters intercept (−μ/*s*) and slope (1/*s*), see Supplementary 3 of Part 1.

#### 2.5.1 Accuracy

We determined the accuracy of the decisions for the treatment options (audiological care and psychosocial counseling) with equations S.2.5a and S.2.5b in Supplementary 2 of Part 1. These are rewritten here for convenience, i.e.


(1a) $$l\,o\,g\,i\,t\,\left(P_{i,j}^{T\,P}\right)=s_{i,j}^{T-}/s_{i,j}^{T+}\cdot l\,o\,g\,i\,t\,\left(P_{i,j}^{F\,P}\right)+2\,\mu_{i,j}/s_{i,j}^{T+},$$

(1b) $$2\,\mu_{i,j}=\mu_{i,j}^{T+}-\mu_{i,j}^{T-}.$$

Where *P^TP^* is the probability of a true positive response for decision *i* and driver *j*. A true positive response is defined as the joint occurrence of a positive truth state T+ and a positive decision, say D+. These probabilities were calculated for audiological care and psychosocial counseling, respectively. Likewise, *P^FP^* is the probability of a false positive response. This is defined as the joint occurrence of a negative truth state T− and a positive decision D+. Again these probabilities were calculated for audiological care and psychosocial counseling, respectively. The logit is the inverse of the cumulative distribution function of the logistic distribution, i.e., logit(z) = ln (z/1-z) with ln denoting the natural logarithm. Equation 1 expresses a linear relation between the logit of the probably of a true positive response and that of a false positive response. The intercept corresponds to the accuracy of the decision process. This is the ability to distinguish between the two truth states and select a decision that agrees. The slope corresponds to the ratio of the square root of the variances of the probability distribution functions of occurrence of the truth states as function of the decision driver (hearing loss or baseline THI-score).

#### 2.5.2 Utility

To quantify utility, we used equation 1 from Part 1. We have rewritten it here in improved form for convenience, i.e.


(2) $$ℒ\,\left(c_{\mathrm{optimal}}\right)=\frac{P\,\left(T-\right)}{P\,\left(T+\right)}\,\frac{\left\{B\left(T-\&D-\right)+C\left(T-\&D+\right)\right\}}{\left\{B\left(T+\&D+\right)+C\left(T+\&D-\right)\right\}}$$

Where ℒ is the likelihood ratio function, which is obtained by dividing the *a posteriori* probability density functions of occurrence of the truth states as function of decision driver. Furthermore, *c*_*optimal*_ is the optimal decision criterion that maximizes expected value, *P*(*T*−) and *P*(*T*+) denote the probabilities of the truth states prior to a decision, B(T+ & D+) and B(T− & D−) are the benefits associated with the joint occurrence of truth states and decisions that agree, and C(T+ & D−) and C(T− & D+) are the costs associated with joint occurrence of truth states and decisions that disagree. The unbiased decision criterion, *c*_*unbiased*_, can be obtained by (numerically) determining the driver value at which the likelihood ratio function equals 1. A decision criterion that corresponds to ℒ > 1 is called strict and a decision criterion that corresponds to ℒ < 1 is called lenient.

#### 2.5.3 Hypothesis testing

We used the “leave-one-out” bootstrapping method to systematically and exhaustively construct different datasets by leaving out a different single case each time. For example, for all participants we created 143 datasets with each dataset containing data of 142 participants by leaving out a different participant each time, Swets et al. (2000). To do this, we used the Matlab function “nchoosek” to create datasets for the whole cohort and datasets for the 6 groups (male/female, < 55/≥ 55 years, and bilateral/unilateral). With the Matlab function “randperm” we also created 100 datasets with random permutations of 72 out of 143 participants (random partition 1) and 100 datasets with the remaining 71 participants (random partition 2). For each dataset we estimated the parameters (μ,*s*) of the logistic distributions of the truth states (*T*+ and *T*−) and calculated the cumulative distribution functions and probability density functions for the combinations of starting a sound enrichment device evaluation period (SEDEP) with hearing loss as driver, and psychosocial counselling uptake (PCU) with baseline THI-score as driver.

Next, we calculated logit(*P^TP^*) and logit(*P^FP^*) from the estimated cumulative distribution functions, and we calculated the values of the parameters slope and intercept in equations 1a and 1b. We also constructed the likelihood ratio function ℒ for each dataset by dividing the estimated probability density functions of *T*+ and *T*−. A division operation may produce very large values of ℒ for some subsets of data when dividing by very small values of the estimated probability distribution of *T*−. Therefore, we removed outliers with the Matlab function “rmoutliers” using the method “percentile.” This function removes elements more than 1.5 interquartile ranges above the upper quartile (75 percent) or below the lower quartile (25 percent).

By plotting the median of logit(*P^TP^*) against the median of logit(*P^FP^*) we created empirical Receiver-Operator-Characteristic (ROC) curves. For each dataset, we also plotted the median of ℒ as function of driver. For all functions and variables, we calculated 2.5 and 97.5 percentiles. This allows hypothesis testing at significance level 5% (two-sided). The following hypotheses were tested independently,

H_0_: 2μ/s^*T*+^ = 0,

H_1_: 2μ/s^*T*+^ ≠ 0,

or

H_0_: s^*T*–^/s^*T*+^ = 1,

H_1_: s^*T*–^/s^*T*+^≠ 1,

We also tested for equality of the parameters slope or intercept for males versus females, < 55 years versus ≥ 55 years, bilateral versus unilateral, and random partition 1 versus random partition 2.

## 3 Results

### 3.1 Descriptive statistics

The final cohort of 143 participants had a mean (SD) age of 56 (13) years, a mean (SD) hearing loss of 61 (27) dB(HL), and a median (IQR) baseline THI-score of 42 (32). It included 62 females (62/143 = 43%). Table 1 shows the breakdown of participants in each subgroup, with the median hearing loss and THI-scores representing the subgroup’s hearing loss and tinnitus related quality of life impact. The proportion of males and females was balanced. In the young subgroup, there were 58 (58/143 = 41%) participants, while bilateral tinnitus was recorded for 69 (69/143 = 48%) of the patients, in both cases also constituting an approximately equal subgroup division. The proportions of participants belonging to populations T− and T+ were approximately balanced for each grouping variable, with the biggest difference between the number of participants in T+ (24/58 = 41%) and T− (34/58 = 59%) in the young subgroup. The number of participants in each population for each group was above or equal to 24 providing an acceptable number of sub-samples for the analysis.

**TABLE 1:** Descriptive statistics for the three categories and subgroups.

Grouping variable	Sex	Age	Laterality
Number of participants	143	143	143
**Subgroup 1**	**Male**	**<55 years**	**Bilateral**
Number of participants (% of all)	81 (57)	58 (41)	69 (48)
Hearing level median (IQR)	60 (41)	47 (30)	55 (43)
Baseline THI score median (IQR)	38 (34)	52 (34)	38 (36)
Number of participants *T*+ (% of subgroup)	36 (44)	24 (41)	32 (46)
Number of participants *T*− (% of subgroup)	45 (56)	34 (59)	37 (54)
**Subgroup 2**	**Female**	**≥55 years**	**Unilateral**
Number of participants	62 (43.3)	85 (59.4)	74 (51.8)
Hearing level median (IQR)	58 (45)	75 (31)	70 (30)
Baseline THI score median (IQR)	47 (30)	38 (30)	42 (28)
Number of participants *T*+ (% of subgroup)	35 (57)	47 (55)	39 (53)
Number of participants *T*− (% of subgroup)	27 (44)	38 (45)	35 (47)

IRQ, interquartile range. *T*− is the population that did not experience an improvement >7 points in THI-score after treatment. *T*+ is the population that did experience an improvement >7 points in THI-score after treatment.

One-way analysis of variance (ANOVA) was conducted to check the effects of subgroups on hearing loss and baseline-THI score. There was a significant effect of age on hearing loss [F(22, 142) = 2.46, *p* = 0.001] and laterality on hearing loss [F(22, 142) = 1.71, *p* = 0.036] at 5% significance level, see Table 2. Older participants generally had 28 dB more hearing loss, while patients with unilateral tinnitus had 15 dB more hearing loss. No differences in hearing loss were found between males and females. For baseline THI-scores, ANOVA revealed no statistically significant differences for any subgroup, see Table 3.

**TABLE 2:** ANOVA hearing level.

One-way analysis of variance: median hearing level per grouping variable					
	**Sum of squares**	**DF**	**Mean squares**	**F**	***P***
Sex	4.26	22	0.194	0.753	0.775
Error	30.9	120	0.257		
Total	35.2	142			
Age	10.7	22	0.488	2.46	**0.001**
Error	23.7	120	0.198		
Total	34.5	142			
Laterality	8.52	22	0.387	1.71	**0.036**
Error	27.2	120	0.227		
Total	35.7	142			

Type III sum of squares. Bold values indicate statistically significant results at the 5% level.

**TABLE 3:** ANOVA baseline THI-score.

One-way analysis of variance: median baseline-THI score per grouping variable					
	**Sum of squares**	**DF**	**Mean squares**	**F**	***P***
Sex	11.2	44	0.254	1.04	0.431
Error	24.0	98	0.245		
Total	35.1	142			
Age	12.2	44	0.278	1.22	0.206
Error	22.3	98	0.227		
Total	34.5	142			
Laterality	11.1	44	0.252	1.00	0.480
Error	24.6	98	0.251		
Total	35.7	142			

Type III sum of squares.

### 3.2 Unequal variance signal detection theory

Tables 4, 5 show the results after bootstrapping for the parameters intercept and slope, respectively. For the datasets for the whole cohort, the intercept for SEDEP and hearing loss is not significantly different from zero and the slope is not significantly different from one. The intercept for PCU and THI-score is smaller than zero, but the slope is not significantly different from one, although it is close to being statistically significantly larger than one. For the random partitions 1 and 2 none of the intercepts differ from zero and none of the slopes differ from one. The intercept represents the accuracy of the decision process, i.e., the ability to distinguish between truth states or in other words between effective treatments and ineffective treatments. A zero intercept with slope one equals chance performance.

**TABLE 4:** Results after bootstrapping for parameter intercept.

Decision and driver	Grouping variable	Median	Lower limit	Upper limit	Number of datasets	Number of outliers
SEDEP and HL	All	−0.297	−0.645	0.278	143	17
	Random partition 1	−0.299	−1.868	2.716	100	25
	Random partition 2	−0.376	−1.729	2.949	100	17
	Male	−**0.979^a^^,^^b^**	−**1.277**	−**0.857**	81	29
	Female	**1.603^a^^,^^b^**	**0.888**	**6.005**	62	16
	< 55 years	0.397	−0.122	0.781	58	9
	≥ 55 years	−0.498	−0.872	0.200	85	14
	Bilateral	−0.196	−0.570	0.093	69	11
	Unilateral	1.74	−0.177	5.611	74	1
PCU and THI	All	−**0.395^a^**	−**0.704**	−**0.03**	143	9
	Random partition 1	−0.255	−1.006	0.459	100	23
	Random partition 2	−0.22	−1.071	0.673	100	27
	Male	−0.892	−4.594	0.507	81	17
	Female	**0.19^a^**	**0.004**	**0.596**	62	17
	< 55 years	0.375	−0.077	1.397	58	17
	≥ 55 years	**0.284^a^**	**0.000**	**0.526**	85	11
	Bilateral	**0.479^a^**	**0.020**	**0.860**	69	5
	Unilateral	−0.64	−1.707	0.281	74	18

SEDEP, starting a sound enrichment device evaluation period; HL, hearing loss; PCU, psychosocial counseling uptake; THI, baseline tinnitus handicap inventory score;

*^a^*intercept is statistically significantly different from 0;

*^b^*intercept is statistically significantly different between subgroups. Bold values indicate statistically significant results at the 5% level.

**TABLE 5:** Results after bootstrapping for parameter slope.

Decision and driver	Grouping variable	Median	Lower limit	Upper limit	Number of datasets	Number of outliers
SEDEP and HL	All	0.926	0.724	1.177	143	17
	Random partition 1	0.957	0.081	3.056	100	25
	Random partition 2	0.896	0.044	3.122	100	17
	Male	**0.384^c^^,^^d^**	**0.134**	**0.556**	81	29
	Female	**1.463^c^^,^^d^**	**1.151**	**4.812**	62	16
	< 55 years	**2.093^c^^,^^d^**	**1.266**	**3.054**	58	9
	≥ 55 years	**0.61^d^**	**0.363**	**1.098**	85	14
	Bilateral	**0.285^c^^,^^d^**	**0.134**	**0.415**	69	11
	Unilateral	**1.853^d^**	**0.916**	**4.091**	74	1
PCU and THI	All	1.941	0.934	2.63	143	9
	Random partition 1	0.381	0.014	1.531	100	23
	Random partition 2	0.471	0.039	2.038	100	27
	Male	**6.753^c^^,^^d^**	**3.251**	**15.143**	81	17
	Female	**0.634^c^^,^^d^**	**0.406**	**0.72**	62	17
	< 55 years	1.578	0.237	3.195	58	17
	≥ 55 years	1.003	0.465	1.235	85	11
	Bilateral	1.307	0.728	2.017	69	5
	Unilateral	2.18	0.702	3.119	74	18

SEDEP, starting a sound enrichment device evaluation period; HL, hearing loss; PCU, psychosocial counseling uptake; THI, baseline tinnitus handicap inventory score;

*^c^*slope is statistically significantly different from 1;

*^d^*slope is statistically significantly different between subgroups. Bold values indicate statistically significant results at the 5% level.

Figure 1 shows the ROC-curves for SEDEP and hearing loss (Figure 1A) and PCU and THI (Figure 1B) for the whole cohort, and partitions 1 and 2. The ROC-curves show how the (logit transformed) probabilities of a TP response and FP response are related when a decision criterion changes from very strict (negative axis values) to very lenient (positive axis values). If the slope equals one, the probabilities of TP and FP responses increase with the same rate. For a slope larger than one, the probability of a TP response increases with a higher rate than the probability of a FP response. This favors a more lenient decision criterion. For slopes smaller than one, the probability of a TP response increases with a lower rate than an FP response. This favors a stricter decision criterion.

Also, the likelihood ratio functions (Figures 1C, D) are plotted. The likelihood ratio expresses the odds of a TP response versus the odds of a FP response. Likelihood ratio functions can be interpreted in different ways. A likelihood ratio larger than one indicates higher odds of a TP response compared to an FP response and consequently corresponds to a strict decision criterion. A strict decision criterion may be appropriate in the following set of circumstances. First, when the *a priori* probability of treatment failure, *P*(*T*−), is higher than the *a priori* probability of treatment success, *P*(*T*+). Second, when costs of a treatment are higher than benefits. Alternatively, it may be inappropriate in the following set of circumstances. First, when the *a priori* probability of treatment failure, *P*(*T*−), is lower than the *a priori* probability of treatment success, *P*(*T*+). Second, when the costs of a treatment are lower than benefits. An inappropriate strict decision criterion results in undertreatment.

A likelihood ratio smaller than one, on the other hand, indicates lower odds for a TP response compared to a FP response and corresponds to a lenient decision criterion. A lenient decision criterion may be appropriate in the following set of circumstances. First, when the *a priori* probability of treatment failure, *P*(*T*−), is lower than the *a priori* probability of treatment success, *P*(*T*+). Second, when costs of a treatment are lower than benefits. Alternatively, it may be inappropriate in the following set of circumstances. First, when the *a priori* probability of treatment failure, *P*(*T*−), is higher than the *a priori* probability of treatment success, *P*(*T*+). Second, when the costs of a treatment are higher than benefits. An inappropriate lenient decision criterion results in overtreatment. An unbiased decision criterion maximizes expected value in case of equal costs and benefits (Swets et al., 2000).

Firstly, the results in Figure 1C show that for the whole cohort, the odds of a positive outcome for SEDEP are at chance level. This may be interpreted as SEDEP being an ineffective treatment. An alternative interpretation may be that the decision criterion is unbiased. As the evaluation period is free of charge and the devices may be returned *ad libitum*, costs and benefits may be perceived as equal. Therefore, an unbiased decision criterion may be considered an appropriate decision criterion for SEDEP. Secondly, Figure 1D shows that for the whole cohort, the odds of a positive outcome for PCU are larger than the odds of a negative outcome. This may be interpreted as PCU being an effective treatment. Alternatively, too strict a decision criterion may have been adopted, possibly as the result of perceived costs being (mistakenly) higher than perceived benefits. Given the evidence that CBT is still the most effective treatment for tinnitus, a strict decision criterion may be considered to be inappropriate and may therefore be associated with undertreatment.

Finally, the results based on the datasets for the random partitions give an impression of the differences that may be observed because of subsampling with randomly chosen equally sized partitions. Please note that for SEDEP and hearing loss the curves for datasets with all participants and with random partitions overlap. This means that subsampling has little effect on parameter estimation. For PCU and THI, on the other hand, the curves are clearly different. Although the parameter values are not statistically significantly different, we must be careful when interpreting results for PCU as differences may be the result of sampling variation.

#### 3.2.1 Sex

**FIGURE 2:**
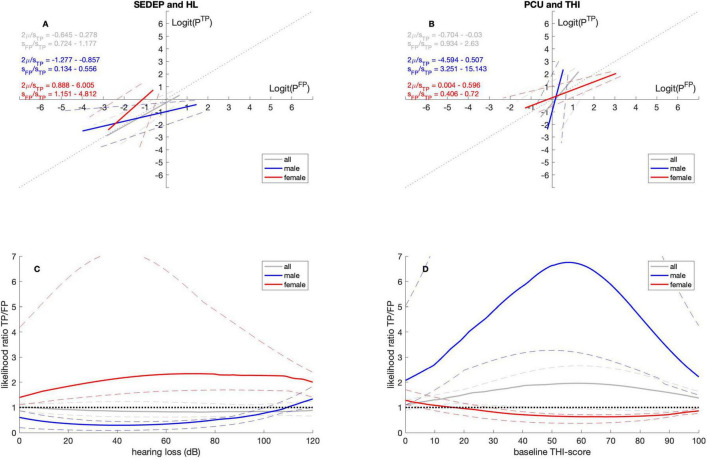
ROC curves **(A,B)** and likelihood ratio functions **(C,D)**. Gray lines for datasets with all participants, blue lines for datasets with male participants and red lines for datasets with female participants. The solid lines denote the medians and the dashed lines the 2.5 percentiles and 97.5 percentiles, respectively. SEDEP, sound enrichment device evaluation period; HL, hearing loss; PCU, psychosocial counseling uptake; THI, tinnitus handicap inventory; TP, true positive; FP, false positive. See text for a discussion.

Tables 4, 5 show that for males and SEDEP the intercept is smaller than zero and the slope is smaller than one. For females the intercept is larger than zero and the slope is larger than one. Also, the intercept and slope for males and females differ statistically significantly. For PCU the intercept differs from zero only for females, being slightly larger than zero. The slope, however, differs from one both for males and females. For males the slope is larger than one and for females it is smaller than one. Again, the slope for males and females differ statistically significantly. Figures 2A, B show the corresponding ROC-curves.

Figure 2C shows the likelihood ratio curves for SEDEP against hearing loss. Please note that for males the likelihood ratio is smaller than one for almost the entire hearing loss range. This indicates that SEDEP may be less effective for males. Alternatively, males seem to adopt a lenient decision criterion regarding SEDEP, possibly leading to overtreatment. For females, on the other hand, the likelihood ratio is larger than one for the entire hearing loss range. Again, this may be interpreted as SEDEP being a more successful treatment for females or, alternatively, females adopting a stricter decision criterion regarding SEDEP, possibly leading to undertreatment. Figure 2D shows that the opposite holds for PCU. Now the likelihood ratio for males is larger than one for all baseline THI-scores. For females, the likelihood ratio is smaller than one for all baseline THI-scores. PCU seems a more successful treatment for males than for females or, alternatively, men adopt a strict decision criterion regarding PCU and females a lenient decision criterion. Consequently, males may be undertreated and females overtreated somewhat.

One explanation for these results might be that males perceive the cost of a positive decision for PCU and a negative outcome as almost 7 times higher than the benefit of a positive decision and a positive outcome. Another explanation might be that males assess the *a priori* probability of a positive outcome *P*(*T*+), as almost 7 times lower than the *a priori* probability of a negative outcome *P*(*T*−). A combination is also possible, of course. Females, on the other hand, may perceive the cost of a positive decision for SEDEP and a negative outcome as twice as high as the benefit of a positive decision and a positive outcome. Alternatively, they may assess the *a priori* probability of a positive outcome as twice as unlikely as the *a priori* probability of a negative outcome.

#### 3.2.2 Age

**FIGURE 3:**
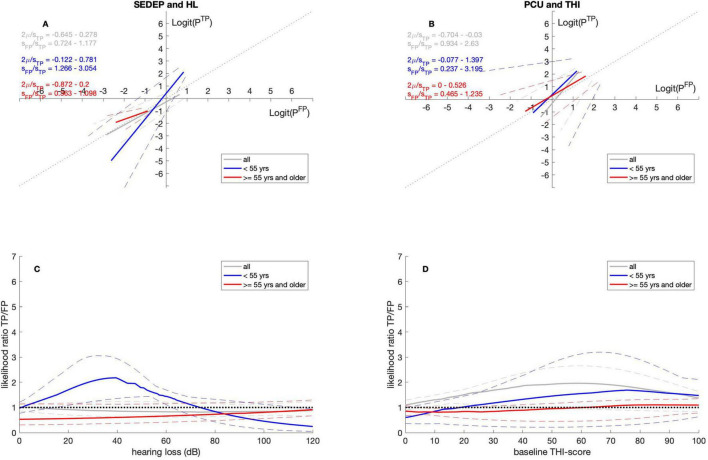
ROC curves **(A,B)** and likelihood ratio functions **(C,D)**. Gray lines for datasets with all participants, blue lines for datasets with participants younger than 55 years and red lines for datasets with participants of 55 years and older. The solid lines denote the medians and the dashed lines the 2.5 percentiles and 97.5 percentiles, respectively. SEDEP, sound enrichment device evaluation period; HL, hearing loss; PCU, psychosocial counseling uptake; THI, tinnitus handicap inventory; TP, true positive; FP, false positive. See text for a discussion.

Tables 4, 5 show that for SEDEP the intercept is not significantly different from zero for both age groups. The slope is larger than one for the under 55 years age group and is not significantly different from one for the age group of 55 years and older. The slope differs significantly between age groups. For PCU the intercept for the under 55 years age group is not significantly different from zero. It is larger than zero for the 55 years and older group. The intercepts do not differ between groups. The slopes for both groups do not differ from one and there is no difference in slope between groups. Figures 3A, B show the ROC-curves. Especially Figure 3B shows that the results for both groups are very similar for PCU.

Figures 3C, D show that the age group of 55 years and older adopts a slightly lenient decision criterion for SEDEP and an unbiased decision criterion or PCU. The age group younger than 55 years adopts a strict decision criterion for SEDEP for hearing losses up to approximately 75 dB(HL) and a lenient decision criterion above. Intuitively, this aligns with higher perceived costs associated with SEDEP as a result of hearing aids being associated with old age. For PCU this group adopts a somewhat strict decision criterion, although it does not reach statistical significance.

#### 3.2.3 Laterality

**FIGURE 4:**
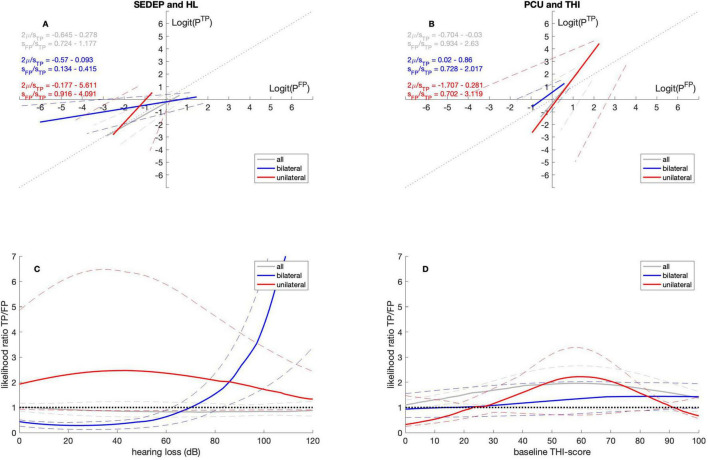
ROC curves **(A,B)** and likelihood ratio functions **(C,D)**. Gray lines for datasets with all participants, blue lines for datasets with participants with bilateral tinnitus and red lines for datasets with participants with unilateral tinnitus. The solid lines denote the medians and the dashed lines the 2.5 percentiles and 97.5 percentiles, respectively. SEDEP, sound enrichment device evaluation period; HL, hearing loss; PCU, psychosocial counseling uptake; THI, tinnitus handicap inventory; TP, true positive; FP, false positive. See text for a discussion.

Tables 4, 5 show that for SEDEP the intercepts do not differ from zero for both the group with bilateral tinnitus and the group with unilateral tinnitus. The slope is smaller than one for the bilateral group and larger than one for the unilateral group. The slopes differ between groups. For PCU the intercept for the bilateral group is larger than zero but does not differ from the intercept for the unilateral group. The intercept for the unilateral group is not significantly different from zero. For both groups the slopes are not significantly different from one and do not differ between groups. Figures 4A, B show the ROC-curves.

Figure 4C shows that for SEDEP and the group with bilateral tinnitus the likelihood ratio is smaller than one for hearing losses below approximately 70 dB(HL) and larger than one above 70 dB(HL). For unilateral tinnitus, the odds of a successful treatment are always higher than the odds of an unsuccessful treatment, indicating an strict decision criterion. Apparently, participants with unilateral tinnitus assess Equation 2 in a way that either the costs of a positive decision and a negative outcome are twice as high as the benefits of a positive outcome, or the *a priori* probability of treatment failure is twice as high as the *a priori* probability of treatment success (or a combination). Figure 4D illustrates that considering PCU, the odds of treatment success and treatment failure do not seem to differ from the results based on the datasets including all participants for both groups. Both groups adopt a somewhat strict decision criterion.

## 4 Discussion

Since multiple courses of action can be taken to treat tinnitus, an aid that can inform patients about the outcome probabilities of treatments with respect to their unique profile would be valuable to both the patient and healthcare professionals designing a treatment plan (Elwyn et al., 2010). Serving this purpose, this study analyzed if, and if so how, the factors sex, age and laterality of tinnitus influence the accuracy and utility of decisions of tinnitus patients for audiological and psychological treatment. The analysis showed multiple effects, indicating that personal characteristics affect treatment decision strategies.

### 4.1 Sex

Females seem to adopt a strict decision criterion for audiological care, while males adapt a lenient decision criterion. This suggests that females evaluate benefits and costs of audiological care differently from males. This is consistent with Güney et al. (2024), who report on general help-seeking behavior, showing that females are less likely to seek help due to societal limitations and their preference for informal sources (e.g., friends and family) of help. The high likelihood of success of psychosocial counseling may indicate that it is more effective for males. On the other hand, it may also indicate that males adapt too strict a decision criterion. This suggests that males cautiously evaluate the necessity of psychosocial counseling, leading to selective engagement, which aligns with Liddon’s report on limited psychological help-seeking behavior from men (Liddon et al., 2018). Hence, when assessing treatment options for tinnitus patients, appreciating a combination of these effects is recommended, including paying attention to promote and normalize psychological help-seeking, considering sex-based differences in treatment efficacy, and tailoring interventions to the individual’s specific baseline characteristics, such as hearing loss severity and baseline THI-scores.

### 4.2 Age

Clear differences were also found between the younger and older subgroups. Younger participants with lower to medium hearing loss adopt a strict decision criterion regarding audiological care, while concerning psychosocial counseling they may utilize a somewhat strict decision criterion over almost all the baseline THI-score range. For younger patients, both treatments can be successful, but hearing loss severity must be considered when deciding between a combined or solely psychological treatment, as hearing loss is less common in this younger age group (Oosterloo et al., 2020). However, if hearing loss is present it might be more severe (Cvorovic et al., 2021). For older participants, on the other hand, there was a clear tendency for an unbiased decision criterion regarding psychosocial counseling for all baseline THI-scores, while audiological treatment was subjected to a somewhat lenient decision criterion resulting in less successful outcomes. This tendency might be because older people have hearing loss that slowly progressed over time, which they have gotten used to. Hence, in the older group slowly progressing hearing loss might not be the primary cause of tinnitus, while for the young the more sudden development of hearing loss is. This is consistent with the findings that aging is independent of the development of tinnitus (Oosterloo et al., 2020). Moreover, this underpins the evidence that tinnitus in patients triggered by idiopathic sudden sensorineural hearing loss is associated with younger age, (Cvorovic et al., 2021).

### 4.3 Laterality

The results showed that in the bilateral group, hearing loss had a bigger effect on the decision of audiological care compared to the unilateral group. Hearing loss by itself is associated with a high probability of tinnitus development (Krauss et al., 2016). Hearing aids help reduce the burden of hearing loss, and consequently by amplifying external sounds masking the tinnitus percept (Vestergaard Knudsen et al., 2010). This might explain the higher success rate of audiological care in case of bilateral tinnitus and hearing loss above 70 dB(HL). In the unilateral group, on the other hand, baseline THI-score was a strong driver for the decision to pursue treatment. The group with unilateral tinnitus benefits more from psychosocial counseling for baseline THI-scores between approximately 25 and 90 points.

### 4.4 Improving SDM practice in tinnitus care

The highest level of evidence of treatment effect provided to patients in SDM to base their decisions on, is obtained from (systematic reviews of) RCTs. These studies generally report mean treatment effects and may provide estimates of the *a priori* success and failure rates of treatments. In circumstances of large treatment effect and small variation between patients, mean treatment effects are very useful to predict the treatment outcome for a particular patient. In case of professional equipoise, treatment effects may be small compared to the interindividual variance. In this case mean treatment effects may not be a good predictor of treatment outcome for a particular patient, as the treatment may still be very effective for the subgroup of patients that this particular patient belongs to. With our model, decision criteria can be determined for different patient groups. Quantifying the costs and benefits that are associated with treatment choices may help to maximize expected value and thus may personalize accuracy and utility of a specific treatment. Ultimately making it possible to aid SDM practices with calculated predictions for treatment outcomes tailored to the patient’s needs.

### 4.5 Methodological considerations

We acknowledge that, regardless that the tinnitus handicap inventory (THI) used in this study is a widely used and well-established questionnaire, some studies on tinnitus use the tinnitus functional index (TFI) instead (Langguth and De Ridder, 2023). In addition, the cutoff for successful treatment (decrease of more than 7 points) has a fundamental effect on the analysis.

Studying patient decisions is not possible within the requirements of a randomized control trial, as it randomly allocates participants. Hence, in our study to assess decisions we used data from clinical practice. This approach inevitably leads to selection bias as our study includes a clinical population of a specific audiological center, leading to outcomes that cannot be directly generalized to the overall population, due to for example the tendency of increased hearing loss in this cohort. Future research should aim to refute or confirm the results presented to ensure robustness across a broader population.

In this study, we looked at the characteristics of sex, age, and laterality in a factor-by-factor analysis, excluding the multifactorial variances (e.g., difference between older and younger females) from the model. We also aim to expand the analysis by mapping the accuracy and utility of the decision for device uptake.

## 5 Conclusion

The aim of the study was to assess the accuracy and utility of decisions about audiological care and CBT-based psychosocial counseling for tinnitus stratified by sex, age and tinnitus laterality. The findings reveal that under the trends for the whole cohort, there are individual variations affected mostly by sex, and to a lesser extent by age and laterality of tinnitus. These findings underscore the importance of personalized approaches in audiological care and psychosocial counseling for tinnitus. Considering individual patient characteristics will enhance treatment efficacy and the quality and effectiveness of healthcare consultations. Future research should expand on these insights by analyzing broader patient populations and refining the predictive model to enhance SDM between patients with tinnitus and their health care professionals, ultimately leading to more suitable and successful treatments.

## Data Availability

The raw data supporting the conclusions of this article will be made available by the authors, without undue reservation.
